# Influence of Selected Crosslinking Agents and Selected Unsaturated Copolymerizable Photoinitiators Referring to the Shrinkage Resistance of Solvent-Based Acrylic Pressure-Sensitive Adhesives

**DOI:** 10.3390/polym14235190

**Published:** 2022-11-29

**Authors:** Adam Licbarski, Marcin Bartkowiak, Zbigniew Czech

**Affiliations:** Department of Chemical Organic Technology and Polymeric Materials, Faculty of Chemical Technology and Engineering, West Pomeranian University of Technology in Szczecin, Piastów Ave. 42, 71-065 Szczecin, Poland

**Keywords:** pressure-sensitive adhesives, crosslinking agents, copolymerizable photoinitiators, shrinkage, PVC film, solvent-based acrylic PSA

## Abstract

The properties of solvent-based pressure-sensitive adhesive (PSA) acrylics, especially shrinkage, are mostly determined by the type and amount of the crosslinking agent added to the prepolymer or by the crosslinking method. The shrinkage profiles of the selected solvent-based acrylic PSA coated on PVC film were investigated using metal chelates (between 0 and 0.55 wt.%), N-methylol acrylamide (up to 8 wt.%), polycarbodiimide and amino resins (up to 6 wt.%), diisocyanate (up to 1 wt.%), multi-functional propylene imines (up to 0.9 wt.%), conventional photoinitiators (up to 3 wt.%) and copolymerizable photoinitiators (up to 2 wt.%). These chemicals were both crosslinking agents that react after the solvent has been evaporated or at higher temperatures, and to the crosslinking agents that react under UV radiation. Some of them were copolymerizable, and others were added to the prepolymer before crosslinking. The best results of shrinkage (0.2%) were obtained by using the UV-crosslinking method and copolymerizable photoinitiators ZLI 3331 and ABP, as well as metal chelates AlACA and TiACA and multifunctional propylene imine Neocryl CX-100 (0.2%). Acceptable results were also achieved for amide BPIA (0.3%), benzophenone derivative PCB (0.4%), N-methylol acrylamide (0.35%) and benzoguanamine resin Cymel 1123 (0.45%).

## 1. Introduction

Since their development several dozen years ago, acrylic pressure-sensitive adhesives have been successfully introduced in many fields of industry. They are used in self-adhesive tapes and labels, marking and protective films, warning or advertising signs and in transdermal dosage systems for pharmaceutical applications, in medical electrodes and medical articles, etc. The technology of acrylic pressure-sensitive adhesives has made remarkable progress over the last sixty years. Thus, many of the largest chemical companies operating in the adhesives market have introduced advanced methods of testing the properties of self-adhesive materials. Properties, such as tack (initial adhesion), adhesion (peel adhesion), cohesion (shear strength) and shrinkage [1,2,3,4,5,6]. These performances are to a large degree determined among others, by the type and amount of the crosslinking agents added to the adhesive and determined by the UV-initiated crosslinking, in the case of photoreactive solvent-based acrylic PSA [1,2,6,7,8,9,10,11,12].

Although the global production of solvent-based acrylic pressure-sensitive adhesives increases year after year, up to now, the lack of comprehensive publications concerning the synthesis, crosslinking and technology of acrylic PSAs can be observed.

Prior to the crosslinking process, the molecules, which build the PSA acrylics, are spaced apart by a distance equal to the van der Waals radius. Following the crosslinking, these intermolecular distances are significantly reduced by the formation of covalent bonds between monomers. Thus, the formation of the desired highly crosslinked PSA material occurs. The reduction of distances between molecules creates internal stress throughout the crosslinked material. This reduces the adhesion of the pressure-sensitive adhesive to the joined surfaces [1,6,13,14,15,16].

Shrinkage of the polymeric materials, including resins or pressure-sensitive adhesives, is a fairly well-known phenomenon. It can be observed when the dimensions of the test bar or plaque do not exactly match the mould from which it was made. The shrinkage takes place, either through the differential thermal expansion between the mould material and the polymer or through microstructural changes in the polymer after moulding. Although polymers based on acrylics are commonly used as pressure-sensitive adhesives in many branches of industry, a property typical for all acrylic PSAs is their shrinkage on PVC surfaces upon crosslinking. It significantly decreases the adhesion performance of the final products [1,3,6,16]. This phenomenon occurs due to the formation of a three-dimensional network structure, during the process of crosslinking. This covalently crosslinked network reduces the intermolecular distances between the monomers used for its formation [1,3,13,17].

The shrinkage of acrylic pressure-sensitive adhesives plays a key role in the production of PVC with self-adhesive films. These types of products are used worldwide in the amounts of billions of square meters as protective films for varnishes in the automotive and furniture industries, as advertising banners and marking films. Two weeks after seasoning at 70 °C, the shrinkage of acrylic adhesives applied to PVC films is tested. Two weeks later, the shrinkage is measured under a special magnifier and given in millimetres (mm) or percentages (%). Generally, a level of 0.5% is acceptable, which allows the use of adhesives with this reduction in the production of decorative and protective self-adhesive films. Shrinkage within 0.3% characterizes the acrylic self-adhesive adhesives of excellent quality [18].

The problem of shrinkage does not only affect PSAs on PVC protective films. The widespread use of PSA in TFT-LCDs or modern OLEDs, including the latest flexible displays, makes it necessary to take this PSA property into account when developing new adhesives [19,20]. Despite the significant advances in display technology, the dimensional stability of the adhesive layers connecting the subsequent parts of the displays is important for their durability. Similar technology for the production of OLED displays has been proposed for encapsulation with the latest technology of organic photovoltaics OPV. The phenomenon of adhesive shrinkage and its influence on the OPV cells was also investigated, in comparison with the design of OLED displays [21].

The phenomenon of PSA shrinkage is often associated with unfavourable operating conditions, e.g., increased temperature. When developing self-adhesive adhesives resistant to high temperatures, the dimensional stability of the adhesive layers is always tested. This applies, inter alia, to silicone self-adhesive adhesives [22,23].

A series of experiments were performed in this work to investigate the influence of the different crosslinkers and photoinitiators on shrinkage after crosslinking. 

The following compounds were tested. Metal chelates—aluminium and titanium acetylacetonate, and other crosslinkers, such as N-methylol acrylamide (N-MAA), policarbodiimide Permutex XR-5580, and amino resins Cymel 303, Cymel 370 and Cymel 1123, isophorone diisocyanate (IPDI), propylene imine crosslinkers N,N′-bis-propylene isophthalic acid amide (BPIA) and trimethylolpropane-tris-(N-methylaziridinyl) propionate (Neocryl CX-100). Furthermore, conventional photoinitiators Irgacure 184, Michler’s ketone (Bis [4-(dimethylamino)phenyl]methanone), multifunctional photoinitiator TBPO (tris-benzophenyloxy phosphineoxide) and photoreactive crosslinker 2,4-bis-trichloromethyl-6-(1-naphthyl)-s-triazine BN-s-T. Copolymerizable photoinitiators, 4-(2-acryloyloxyethoxy)phenyl-2-hydroxy-2-propyl ketone (ZLI 3331), 4-acryloyloxy benzophenone (ABP), 4-acrylamidocarbonyldioxy benzophenone (ACDP), 4-benzophenyl vinyl carbonate (BVCN) and additionally, 4-propylene imine carbonyl benzophenone (PCB) were also evaluated.

The following concentrations of the selected crosslinking agents and copolymerizable photoinitiators were used: metal chelates (between 0 and 0.55 wt.%), N-methylol acrylamide (up to 8 wt.%), polycarbodiimide and amino resins (up to 6 wt.%), diisocyanate (up to 1 wt.%), multi-functional propylene imines (up to 0.9 wt.%), conventional photoinitiators (up to 3 wt.%) and copolymerizable photoinitiators (up to 2 wt.%).

The given concentrations of the cross-linkers and photoinitiators were based on both our team’s current work experience at the West Pomeranian University of Technology Szczecin, and on the author’s (Z.C.) own experience during his previous work at Lohmann, UCB and Chemitec. Typical crosslinkers and conventional saturated photoinitiators were added to the pressure-sensitive adhesives after the polymerization process, while the unsaturated photoinitiators were built into the polymer chain during the polymerization. The crosslinking of adhesives containing conventional, as well as unsaturated polymerizable photoinitiators, occurs under a UV lamp. The concentration of the crosslinkers and the concentration of photoinitiators refer to the polymer content of the solvent-based acrylic PSA. The studied crosslinking agents react with the carboxyl groups derived from acrylic acid, incorporated into the polymer backbone.

## 2. Materials and Methods

### 2.1. Synthesis of the Investigated Solvent-Based Acrylic Pressure-Sensitive Adhesives

The solvent-based acrylic PSAs for the study were synthesized in the presence of 0.1 wt.% of AIBN (the radical initiator) with 50 wt.% of polymer contents and the environment of ethyl acetate at 78 °C. The dosage time of the monomers mixture was equal to 2 h and the post-reaction time was 5 h. The monomers mixture consisted of 2-ethylhexyl acrylate, butyl acrylate and acrylic acid in the proportions of 60:35:5 [wt.%]. Following the polymerization, the above-mentioned crosslinkers were added, in different concentrations, to the synthesized acrylic PSA. All of the monomers, as well as ethyl acetate and AIBN, were obtained from BASF (Ludwigshafen, Germany).

### 2.2. Viscosity and Molecular Weights (M_W_ and M_n_) of the Prepared Acrylic PSAs, including Photoreactive Acrylic PSA Containing Unsaturated Photoinitiators

The viscosity of the investigated solvent-based acrylic pressure-sensitive adhesives was determined using the Rheomat RM 189 apparatus (Rheometric Scientific GmbH, Munich, Germany), with spindle No. 3, at temperature of 23 °C.

The studies of the molecular weights were performed in THF, using LaChrom LC system equipped with a RI Detector L-7490 and LaChrom UV Detector L-7400 from Merck Hitachi (Hitachi Europe GmbH, Düsseldorf, Germany) using a PLgel 10^6^ Å column from Hewlett-Packard (Agilent Tech., Waldbronn, Germany).

### 2.3. Amount of Solid Materials

The amount of solid residues was determined by weight after drying the samples for 45 min at 140 °C, using a moisture analyzer (Radwag MA 110.R, Radom, Poland. The residuals of the monomers were measured with a gas chromatograph Trace GC 2000 chromatograph (Thermo-Finnigan, Thermo Fisher Sci., Warsaw, Poland), equipped with a J&W DB-1 column and a FID detector.

### 2.4. The Coating Weight of the Acrylic PSA

The coating weight of the pressure-sensitive adhesive after the organic solvent removal (thickness of the adhesive layer) has a significant influence on the shrinkage. The basis weight of the adhesive layer covering the foil was maintained at 60 g/m^2^.

### 2.5. Type of Carrier, Drying Terms in Drier and Cover Material

Throughout the study, the adhesives were coated on the 95 g/m^2^ siliconized paper from Laufenberg (Krefeld, Germany), dried for 10 min at 110 °C to be free from the polymerization solvents and after that, transferred on 90 g/m^2^ PVC film containing plasticizer Hexamoll Dinch (BASF, Ludwigshafen, Germany). The self-adhesive PVC films were placed on the aluminium plates.

### 2.6. UV-Crosslinking

The final photoreactive samples containing saturated and unsaturated photoinitiators, in the form of dried adhesive layers with a 60 g/m^2^ coat weight, were prepared using a UV-lamp U350-M-I-DL (IST METZ, Nürtingen, Germany) with a UV-A wavelength between 315 to 380 nm and UV-dosage of about 100 mJ/cm^2^ after a 120 s exposure time. The UV exposure was measured by integrating the radiometer UV Power Puck^®^ II, available from EIT2.0 (Leesburg, VA, USA)

### 2.7. Conditioning

The adhesive-coated strips were stored for one week at room temperature and at a relative humidity equal to 50%, before the tests were carried out. Three samples were tested subsequently and the final value of the shrinkage was the arithmetic mean of the achieved single results.

### 2.8. Evaluation of the Shrinkage

The shrinkage was evaluated, according to the modified standard FINAT FTM 14 (Dimensional stability) [24]. The method of measurement involves sticking the sample self-adhesive PVC films to aluminium plates with defined dimensions (100 mm × 100 mm). Two straight perpendicular lines were then made on every sample with a sharp knife, making a cross-shaped incision. The samples were then aged for two weeks in the conditioning oven at 70 °C. Next, the changes in the width of the slit cut on the foil were checked at eight different points and the result for every sample was the arithmetic mean of eight measurements.

Shrinkage presents the change of dimensions (as a percentage or metric value) of the PVC foil covered with PSA after its crosslinking, and attached to the glass or metal plate. For the shrinkage greater than 0.5% or l_r_ greater than 0.5 mm, other properties of the sample were neglected.

### 2.9. Chemical Substances Used in the Research

The crosslinking agents used in the study are presented in Table 1. All investigated reagents were used as-received without further purification.

## 3. Results and Discussion

### 3.1. Viscosity and the Molecular Weights (MW and Mn) of the Synthesized Acrylic PSAs, including Photoreactive Acrylic PSAs Containing Unsaturated Photoinitiators

The solvent-based acrylic PSA was characterized by the following parameters: viscosity of 9.1 Pa·s, the weight average molecular weight MW of 825.000 Da, the number average molecular weight Mn of 280.000 Da and polydispersity Pd of 2.95 (MW/Mn). The incorporation of unsaturated photoinitiators ZLI 3331, ABP, ACDP and BVCN in the concentration up to 2.0 wt.% slightly increases the viscosity and both molecular weights MW and Mn.

### 3.2. Shrinkage of the Investigated Solvent-Based Acrylic PSAs

The main target of the presented investigations was the development of solvent-based acrylic PSAs, which could be applied for the polyvinyl chloride (PVC) sign and marking films with a low shrinkage and high performance. The greatest attention was paid to the shrinkage parameter. For the samples with shrinkage greater than 0.5%, other properties (tack, peel adhesion and shear strength) were neglected.

The solvent-borne acrylic pressure-sensitive adhesive formulations, containing selected crosslinking agents or selected photoinitiators, were cast with a knife coater on the siliconized paper with 60 g/m^2^ dry adhesive coat weight, dried 10 min at 110 °C, or by using thermal crosslinking agents for 10 min at 140 °C, and after drying applied onto the PVC film. In the case of tested photoinitiators, the coated adhesive layer on siliconized paper was UV-crosslinked with a UV lamp before the application on the PVC film took place. The effect of the UV crosslinking by using a UV lamp on the shrinkage of acrylic PSAs for the selected types of photoinitiators, was evaluated at 100 mJ/cm^2^ UV dose and for a 2 min UV-crosslinking time.

**Figure 1:**
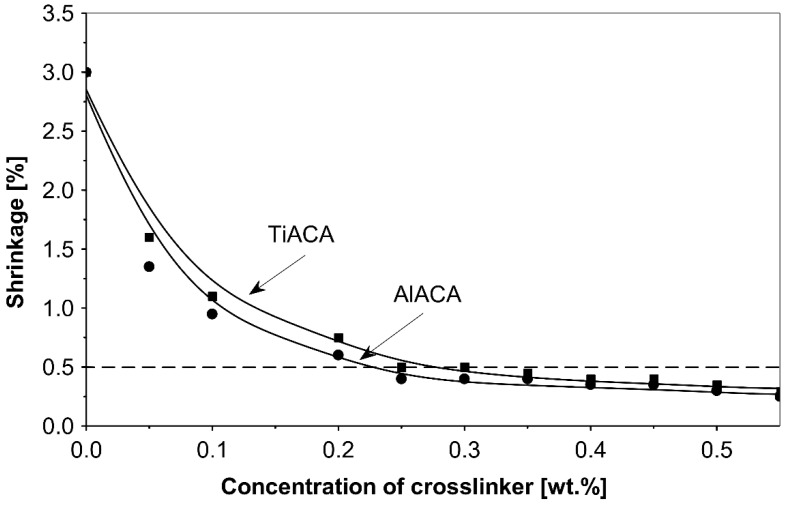
Shrinkage of the acrylic PSAs crosslinked with selected metal acetylacetonates AlACA and TiACA.

The influence of the concentration of the external crosslinkers aluminium acetylacetonate and titanium acetylacetonate on the shrinkage of the synthesized solvent-borne acrylic pressure-sensitive adhesives containing AlACA and TiACA, is presented in Figure 1.

As could be expected, the increase in the amount of the metal chelates crosslinking agents AlACA and TiACA results in the decreased shrinkage of acrylic PSAs. A better shrinkage resistance was achieved using AlACA, compared to TiACA. It is the main reason for more and more replacing TiACA with AlACA as a crosslinking agent in the technology of solvent-based acrylic PSAs. Below the concentration of 0.2 wt.% of AlACA or 0.2 wt.% of TiACA, the shrinkage values of 0.6% and 0.75% were not acceptable. Above 0.2 wt.% of both metal acetylacetonates, the shrinkage level dropped under 0.5%.

**Figure 2:**
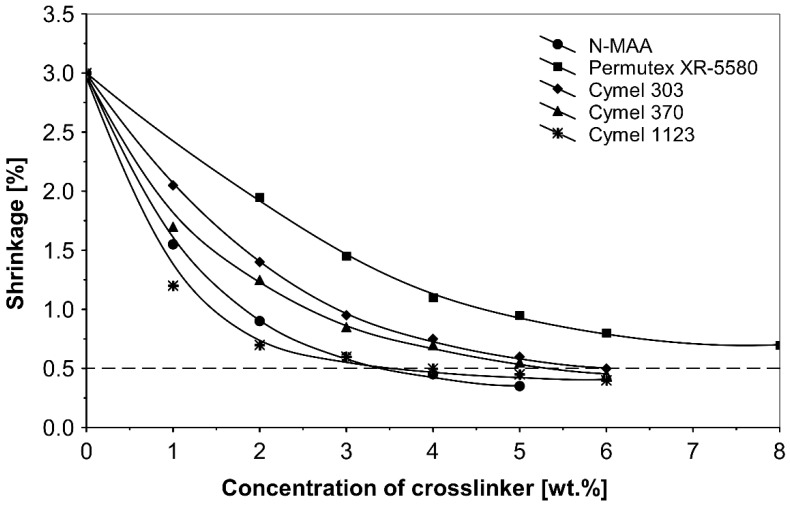
Shrinkage of the acrylic PSAs crosslinked using: N-MAA, Permutex XR-5580 and selected Cymel amino resins.

The shrinkage profiles of the solvent-based acrylic PSAs crosslinked by use of different crosslinkers, such as N-methylol acrylamide (N-MAA), policarbodiimide Permutex XR-5580 and amino resins: Cymel 303, Cymel 370 and Cymel 1123 are presented in Figure 2.

The best shrinkage results of 0.35% and 0.45% were achieved for 5.0 wt.% N-methylol acrylamide (N-MAA) and 5.0 wt.% methoxymethyl ethoxymethyl benzoguanamine Cymel 1123. Completely unacceptable, were the shrinkage runs for acrylic PSA crosslinked with polycarbodiimide Permutex XR-5580.

The observed differences result mainly from the structure of these compounds and the number of functional groups, which react with carboxylic groups of the polymer chain. N-MAA is the monofunctional internal crosslinker, and built-in the polymer structure. Cymel 1123 is the benzoguanamine derivative and it has four-functional, in comparison to six-functional melamine derivatives (Cymel 303 and 370). The structure of this compound, including the presence of the phenyl group, has a positive effect on the dimensional stability during the crosslinking. Permutex XR-5580 is a polymer containing a large number of polycarbodiimide functional groups, that react during the crosslinking with the polymer chain, resulting in a much higher shrinkage of the adhesive layer.

**Figure 3:**
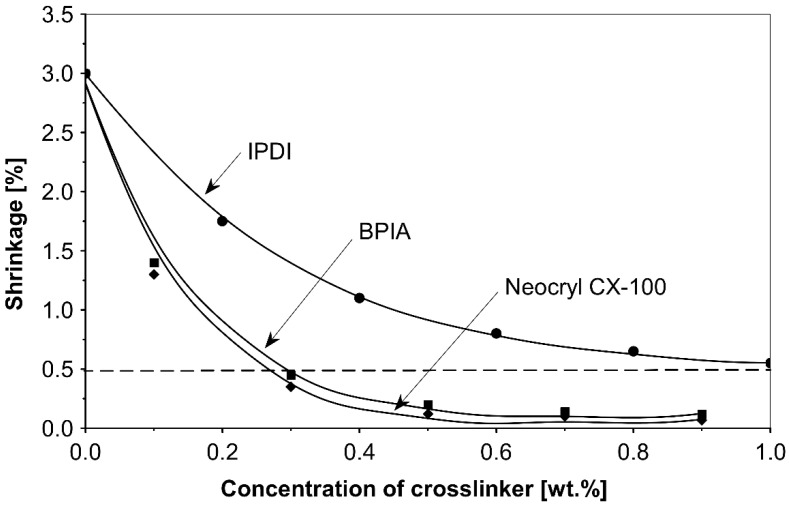
Shrinkage of the acrylic PSAs crosslinked using IPDI, BPIA and Neocryl CX-100.

In Figure 3, the influence on shrinkage of the acrylic PSAs is shown for isophorone diisocyanate (IPDI) and propylene imine crosslinkers N,N′-Bis-propyleneisophtalic acid amide (BPIA) and trimethylolpropane-tris-(N-methylaziridinyl)-propionate (Neocryl CX-100), which are usually typical for these types of crosslinkers, with concentrations between 0.1 wt.% and 0.9 wt.%.

It can be seen that the lowest values of shrinkage were achieved using the tested propylene imines as crosslinkers in a concentration above 0.2 wt.%. An excellent shrinkage level of 0.2% was observed using 0.5 wt.% of Neocryl CX-100. The shrinkage, measured for acrylic PSAs, crosslinked with IPDI was entirely unacceptable, in comparison to the selected propylene imine. At first, this seems surprising but can be explained by an influence of a higher reactivity of the propylene imine derivatives, compared to the multifunctional isocyanates in reaction with the carboxylic groups of acrylic acid in the acrylic polymer chain. As an indication of the reactivity of the crosslinker in the presence of the carboxylic groups, the pot life of the acrylic self-adhesives containing acrylic acid in the polymer structure is taken. The pot life of the solvent-borne acrylic PSA systems containing multifunctional isocyanates, is short and ranges between 8 to 24 h. For similar PSA systems, containing multifunctional propylene imine, the pot life is still shorter and is limited to several hours. With the increasing propylene imine crosslinker content, there is a strong decrease in the acrylic PSA pot life.

**Figure 4:**
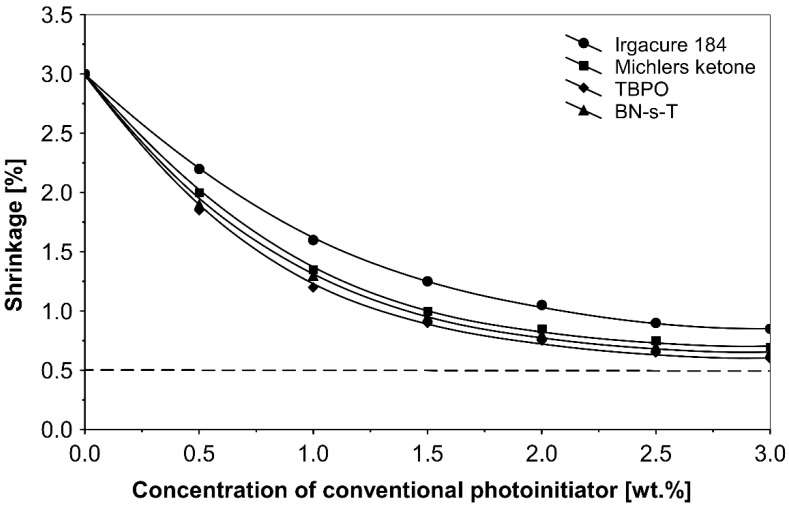
Shrinkage of the acrylic PSAs containing selected conventional saturated photoinitiators after the UV crosslinking using a UV lamp.

Figure 4 illustrates the shrinkage characteristics of the solvent-borne acrylic PSAs, containing monofunctional conventional photoinitiators: α-cleavage photoinitiator 1-hydroxycyclohexyl acetophenone (Irgacure 184), H-abstractor Bis[4-(dimethylamino)phenyl]methanone (Michler’s ketone), multifunctional photoinitiator Tri-benzophenyloxy phosphineoxide (TBPO) and photoreactive crosslinker 2,4-bis-trichloromethyl-6-(1-naphthyl)-s-triazine (BN-s-T), after the crosslinking processes initiated using a UV lamp.

Shrinkage studies have proved that the evaluated photoinitiators exhibited, at increased concentrations, unacceptable shrinkage values (>0.6%). The best shrinkage performance of 0.6% was achieved with acrylic PSA containing the multifunctional photoinitiator TBPO.

**Figure 5:**
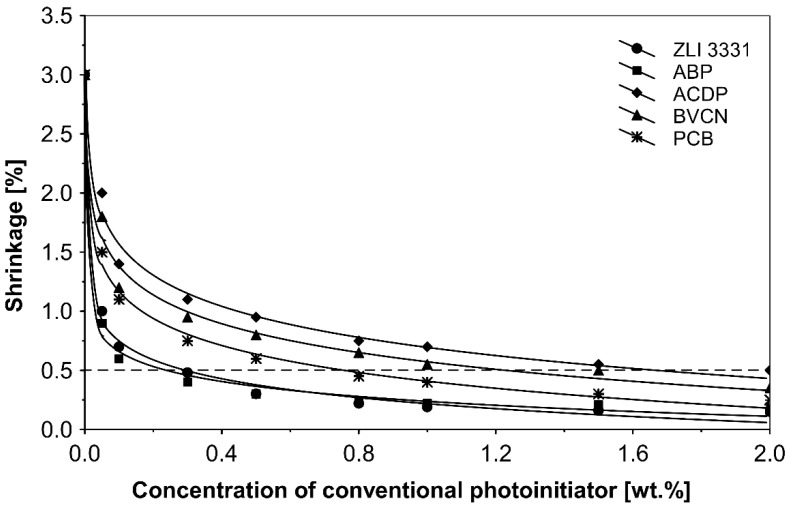
Shrinkage of the acrylic PSAs containing copolymerizable and the additional photoinitiators after the crosslinking using UV radiation.

Figure 5 is a graph showing the effect on the shrinkage of acrylic pressure-sensitive adhesives containing varying amounts of five different photoinitiators: acryloyloxy α-cleavage photoinitiator 2-hydroxy-1-[4-(2-acryloyloxy)phenyl]-2-methyl-1-propanone (ZLI 3331), copolymerizable H-abstractor 4-acryloyloxy benzophenone (ABP), unsaturated 4-acrylamidocarbonyldioxy benzophenone (ACDB), 4-benzophenylvinyl carbonate (BVCN) and the additional 4-propyleneiminecarbonyl benzophenone (PCB).

The results show that all selected investigated unsaturated photoinitiators tend to generate overall a good shrinkage resistance. It is worth paying attention to the fact that the shrinkage of solvent-based acrylic pressure-sensitive adhesives after UV exposure significantly decreases, suggesting a continued crosslinking activity under UV exposure. Excellent shrinkage values of 0.2% were achieved using ABP and ZLI 3331 for 2.0 wt.% of both photoinitiators. Above 0.3 wt.% ZLI 3331 and 0.3 wt.% ABP, the shrinkage values correspond to less than 0.45% and 0.3%. This has proved that ZLI 3331 and ABP are good alternatives to other photoinitiators (saturated and unsaturated). The best of copolymerizable photoinitiators, due to the very low shrinkage of UV-crosslinked solvent-borne acrylic PSAs, was 4-acryloyloxy benzophenone (ABP).

## 4. Conclusions

The study examined a very large group of crosslinking agents used in solvent-based acrylic pressure-sensitive adhesives, in terms of their influence on the dimensional stability of adhesive layers coated onto PVC surfaces. Various crosslinking agents and various crosslinking methods (commonly used in the PSA industry) were evaluated. The aim was to select a group of crosslinking systems that would best provide stable self-adhesive layers for use in various application conditions. Other performance characteristics of PSA samples (peel adhesion, shear strength and tack) were not compared; this has been the subject of previous studies. The following conclusions can be drawn from the presented results.

The best results were obtained using metal chelates and propylene imines for the group of internal and external crosslinking agents. The best results of shrinkage were observed for aluminium acetylacetonate (AlACA) and amongst the multifunctional propylene imines for trimethylolpropane-tris-(N-methylaziridinyl)-propionate (Neocryl CX-100). By using AlACA as an external crosslinker at a concentration of 0.55 wt.%, the shrinkage was reduced to 0.25%. Moreover, the use of Neocryl CX-100 as an internal crosslinker at a concentration of 0.5 wt.%, resulted in a shrinkage of 0.2%. The use of BPIA, N-MAA and Cymel 1123 also gave quite good results in this group of compounds—0.3%, 0.35% and 0.45%, respectively.

From the experimental results by the application of the UV crosslinking method, the conclusion can be inferred that the use of 4-acryloyloxy benzophenone (ABP), as well as 4-(2-acryloyloxyethoxy)phenyl-2-hydroxy-2-propyl ketone (ZLI 3331), influences positively the shrinkage resistance of the UV-crosslinked acrylic PSAs. For the concentration of both crosslinkers to 2 wt.%, the shrinkage values did not exceed 0.2%.

Conventional photoinitiators, such as Irgacure 184, Michler’s ketone, TBPO and BN-s-T failed to achieve satisfactory results. The best shrinkage values obtained during the experiments were in the range of 0.6–1.0%.

Combining the acrylic PSAs containing ABP or ZLI 3331 as a firm incorporated part of the polymer backbone and using an excimer-laser as a UV crosslinking source, allows an excellent shrinkage performance.

## Figures and Tables

**Table 1:** Overview of the crosslinking agents used in the study.

Raw Material	Abbreviation	Chemical Formula	Supplier	Type *
aluminium acetylacetonate	AlACA	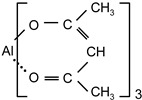	Wacker Chemie Germany	EC
titanium acetylacetonate	TiACA	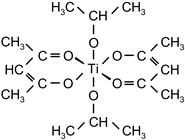	Union Carbide USA	EC
N-methylolacrylamide	N-MAA	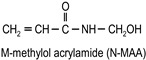	BASF Germany	IC
modified polycarbodiimide	Permutex XR-5580	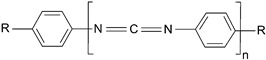	Stahl Chemistry Netherland	EC
highly methylated amine resin	Cymel 303	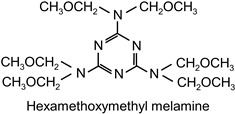	Cytec Netherlands	EC
partially methylated amine resin	Cymel 370	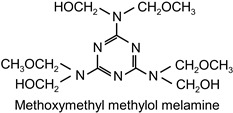	Cytec Netherlands	EC
highly alkylated benzoguanamine resin	Cymel 1123	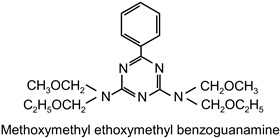	Cytec Netherlands	EC
3-isocyanatemethyl-3,5,5- trimethyl-cyclo-hexylene diisocyanate	IPDI	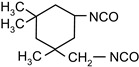	BASF Germany	EC
N,N′-bis-propylene isophthalic acid amide	BPIA	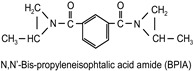	self-synthesized	EC
trimethylolpropane-tris-(N-methylaziridinyl) propionate	Neocryl CX-100	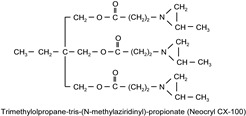	ICI USA	EC
1-hydroxycyclohexyl acetophenone	Irgacure 184	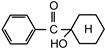	Ciba Swiss	EPh
Bis[4-(dimethylamino)phenyl]methanone	Michler‘s ketone	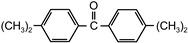	BASF Germany	EPh
tris-benzophenyloxy phosphineoxide	TBPO	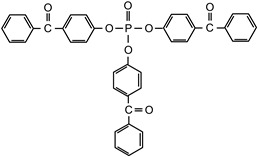	BASF Germany	EPh
2,4-bis-trichloromethyl-6(1-naphthyl)-s-triazine	BN-s-T	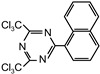	self-synthesized	EPh
2-hydroxy-1-[4-(2-acryloyloxyethoxy)phenyl]-2-methyl-1-propanone	ZLI 3331	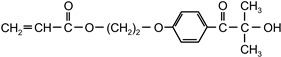	Merck Germany	CoPh
4-acryloyloxy benzophenone	ABP	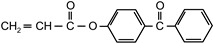	Poly-Chem Germany	CoPh
4-acrylamidocarbonyl-dioxy benzophenone	ACDP	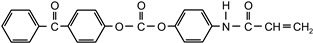	self-synthesized	CoPh
4-benzophenylvinyl carbonate	BVCN	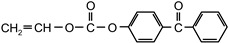	self-synthesized	CoPh
4-propyleneimine-carbonyl benzophenone	PCB	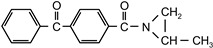	self-synthesized	CoPh

*—EC (external crosslinker); IC (internal crosslinker); EPh (external photoinitiator); CoPh (copolymerizable photoinitiator).

## Data Availability

The raw/processed data required to reproduce these findings cannot be shared at this time as the data also forms part of an ongoing study.
